# Study on the application of King’s combined uterine suture for hemostasis during cesarean section

**DOI:** 10.1186/s12884-021-04231-4

**Published:** 2021-11-10

**Authors:** Li Xia, Jinxiao Lin, Yan Dai, Xinrui Wang

**Affiliations:** 1 Department of Obstetrics, Fujian Maternity and Child Health Hospital, Affiliated Hospital of Fujian Medical University, Fuzhou, 350001 Fujian China; 2 Medical Research Center, Fujian Maternity and Child Health Hospital, Affiliated Hospital of Fujian Medical University, Fuzhou, 350001 Fujian China; 3 Fujian Key Laboratory of Women and Children’s Critical Diseases Research, Fujian Maternity and Child Health Hospital, Affiliated Hospital of Fujian Medical University, Fuzhou, 350001 Fujian China

**Keywords:** Placenta previa, Uterine scarring, Suture technique, Hemostasis, Cesarean section

## Abstract

**Introduction:**

Postpartum hemorrhage is a serious complication of childbirth and is still the leading cause of maternal death worldwide. Lower uterine segment hemorrhage during cesarean section is an important cause of postpartum hemorrhage. Our objective is to expore the efficacy and safety of King’s combined uterine suture for hemostasis during cesarean section.

**Methods:**

We examined 48 cases: 16 cases of pernicious placenta previa (including one case of twins), 11 cases of central placenta previa (including one case of twins), 18 cases of uterine scarring (including two cases of twins), as well as one case of twin pregnancy, two cases of breech presentation, and one case of pulmonary hypertension. The “King’s combined uterine suture” method for hemostasis was used in patients with lower uterine segment hemorrhage during cesarean section.

**Results:**

The results showed that all patients had successful hemostasis during surgery, and there were no cases of hysterectomy.

**Conclusion:**

We have concluded that King’s combined uterine suture is a fast and safe hemostasis method for cesarean section that can effectively reduce blood loss and restore the normal shape of the lower uterine segment. Furthermore, this suture method can reduce postpartum hemorrhage and hysterectomy rate, as well as improve maternal prognosis.

**Supplementary Information:**

The online version contains supplementary material available at 10.1186/s12884-021-04231-4.

## Introduction

The traditional definition of postpartum hemorrhage is at least 500 ml of total blood loss after delivery of the fetus by vaginal birth, or at least 1000 ml of blood loss within 24 h of cesarean birth [1, 2]. Postpartum hemorrhage is a serious complication of childbirth and remains the leading cause of maternal deaths globally, with about 10 maternal deaths per hour due to postpartum hemorrhage [3, 4], and the leading cause of maternal deaths in China [2]. Most pregnancy-related deaths are preventable, including postpartum hemorrhage [5]. Research on postpartum hemorrhage is ongoing, and the postpartum hemorrhage guidelines (2016 edition) published by the Royal College of Obstetricians and Gynecologists (RCOG) [6] and the postpartum hemorrhage guidelines (2017 edition) published by the American College of Obstetricians and Gynecologists (ACOG) [7] both provide detailed descriptions for the prediction, prevention, and treatment of postpartum hemorrhage, as well as the corresponding measures to determine the etiology of postpartum hemorrhage. Primary treatment involves the use of uterotonic medications, bimanual massage of the uterus, and other treatments. If these treatments are ineffective, other treatments, such as uterine packing, uterine artery embolization, uterine artery ligation, uterine compression suture, and even hysterectomy, should be applied in a timely manner.

In the past 12 years, we have extensively applied parauterine vascular ligation for intraoperative hemostasis during cesarean section, with the purpose of controlling bleeding to within 1000 ml of blood loss to prevent postpartum hemorrhage. However, in long-term practice, it has been found that some cases still exhibit diffuse errhysis in the lower part of the uterus after parauterine vascular ligation. For this reason, we tried a new conservative surgical method for hemostasis during cesarean section, King’s combined uterine suture, which involves a combination of parauterine vascular ligation with longitudinal suture of the lower uterine segment. After more than 3 years of clinical application, initial results demonstrate that postpartum hemorrhage can be successfully controlled, especially in cases of pernicious placenta previa, uterine scarring, and lower uterine segment hemorrhage.

## Materials and methods

### Source

Forty-eight inpatient cases were collected from the Second Department of Obstetrics and Maternity of Fujian Maternal and Child Care Service Center from June 2016 to July 2019. The "King's combined uterine suture" method was adopted for lower uterine segment hemorrhage during the cesarean section.

The application of this technology was approved by the Ethics Committee of Fujian Maternal and Child Care Service Center, and all patients and/or their close relatives signed a written informed consent before surgery.

### Methods

#### King's combined uterine suture surgical process.

The cesarean section was completed by the same surgical team. The “King’s combined uterine suture” was the preferred surgical method. During the operation, the puerpera lay down with limbs outstretched in a “starfish” position. Autologous blood was collected and filtered during the operative field hemorrhage, and vaginal bleeding was collected under the hip to accurately calculate the total amount of bleeding. Longitudinal incision or transverse incision was used in the middle of the lower abdomen. For patients with placenta previa, the placental range and fetal position was judged again during the operation, the anterior placenta was palpated and found to be thin and weak, and a uterine incision was designed to avoid the anterior placenta.

**Fig. 1:**
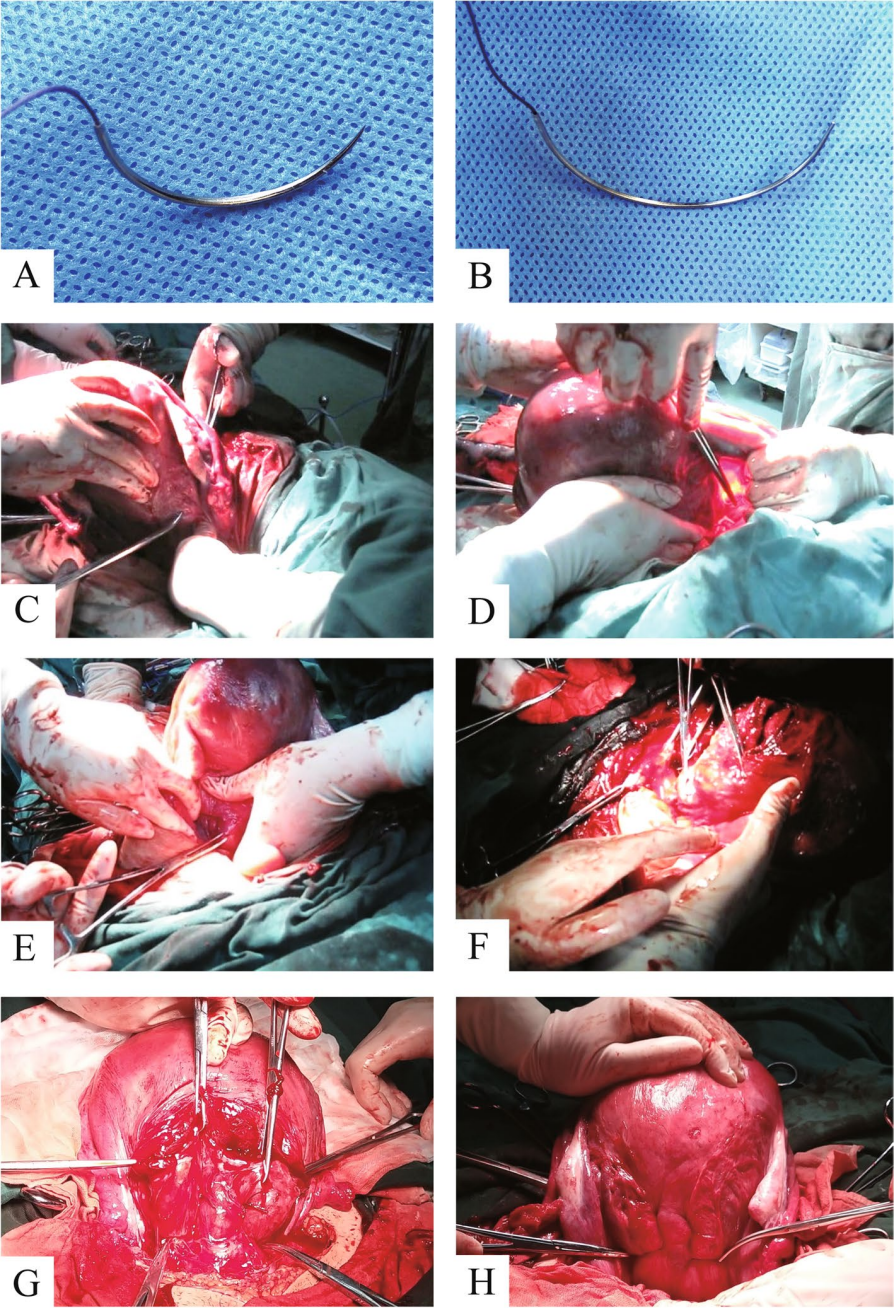
**A** and **B** The needle and thread used in King’s combined uterine suture procedure. **C** and **D** Parauterine vascular ligation (right side view). **E** and **F** Longitudinal suture of the lower uterine segment (left side view). **G** and **H** King’s combined uterine suture (front view and back view)

The parauterine vascular ligation surgery method was performed as follows: After placental expulsion, the uterus was lifted out of the abdominal incision. In cases involving pernicious placenta previa, the placenta was lifted out of the uterus outside the abdominal incision before placental expulsion. Fingers were used to touch the cervical internal opening in the uterine cavity. The bladder was pushed down to the level of the vaginal fornix at the cervical internal opening, which is the indicated point, exposing the upper part of the cervical vagina. Using 358 or 3709 absorbable thread (Johnson & Johnson company), a large needle was inserted from front to back into the cervical muscle layer 1 to 2 cm inside the cervix (see Fig. 1A and B), and then passed through the parauterine vessel from the lateral side after passing through the muscular layer. The needle was pulled from the back to the front of the avascular zone of the broad ligament, and then the parauterine vessel was ligated (see Fig. 1C and D).

**Fig. 2:**
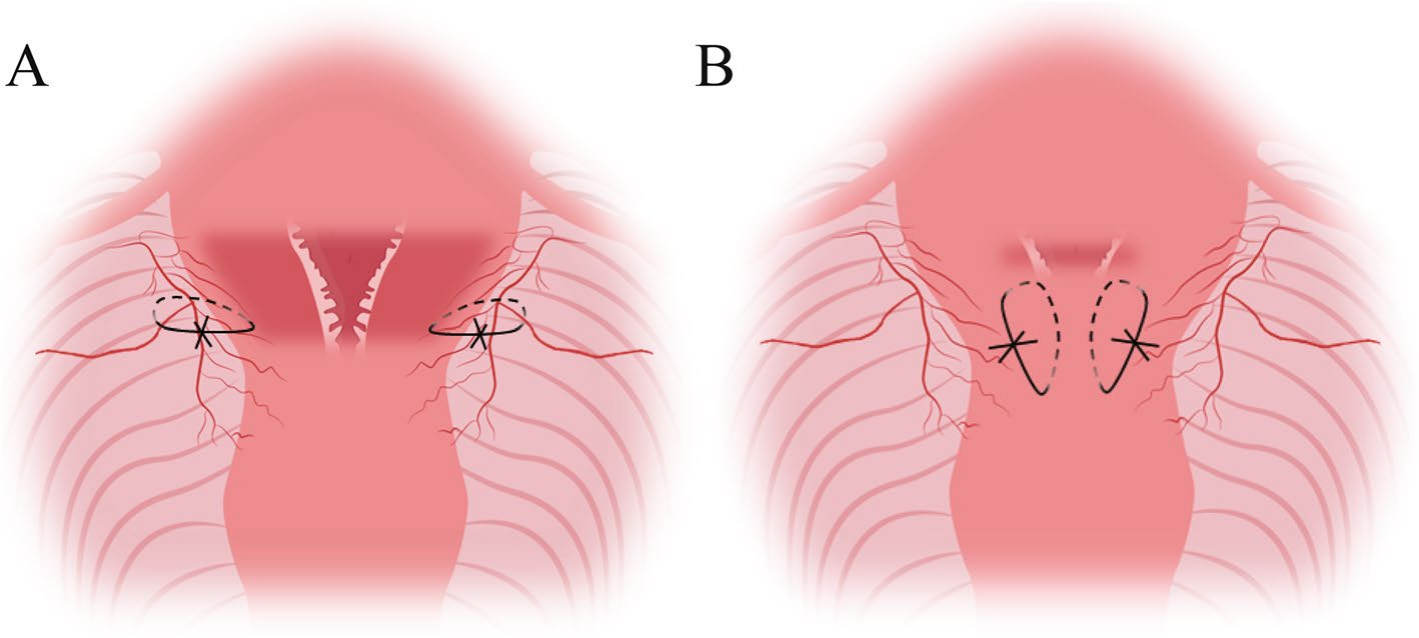
Schematic illustration of King’s combined uterine suture. **A** Parauterine vascular ligation. **B** Longitudinal suture of the lower uterine segment

Longitudinal suture of the lower uterine segment was performed as follows: Based on the parauterine vascular ligation, the same needle line was ligated at the side of the parauterine vessel, 1–2 cm from the outer edge of the lower segment, and ran through the anterior and posterior walls of the uterus, from front to back of the full layer. The lower segment of the posterior wall of the uterus was 3–4 cm from the lateral edge and penetrated the posterior wall of the uterus from the back to the front. The needle was drawn out from the uterine cavity within visible range under the cesarean section incision. The same needle was placed at the corresponding position of the lower segment of the uterine anterior wall, 1 cm from the lower edge of the incision, pushed through the lower segment of the uterine anterior wall, and the uterus was tied to the lower side of the anterior wall. The lower part of the contralateral uterus was treated the same (Fig. 1E, F). Anterior and posterior view of the uterus after King’s combined uterine suture (Fig. 1G, H). A schematic diagram simulated picture of the King’s combined uterine suture is shown in Fig. 2A and B, and an additional movie file shows this in more detail [See Additional file 2].

#### Intraoperative hemostasis supplement program

All cases were routinely administered with 100 units of carbetocin by intravenous injection after delivery. After the King’s combined uterine suture process, there was no active bleeding, but there was still a small amount of errhysis in part of the uterus. Hemorrhage was treated using an “8” suture, bilateral uterine and ovarian artery ligation, and the Hayman suture.

#### Intraoperative and postoperative detection indicators

The amount of intraoperative blood loss, the results from the routine blood test taken on the second day after surgery, and the length of stay in the obstetric intensive care unit (OICU) after surgery were recorded. All patients were followed-up with an ultrasound review of the uterine involution status, 6 weeks after discharge. Follow-up was performed every 3 months in the first year after surgery, and then annually to assess the effectiveness of the King’s combined uterine suture method. Follow-up data were updated by telephone survey before publication.

#### Intraoperative blood loss

Total intraoperative blood loss was measured by summing the amount of blood obtained from a suction device used to collect bleeding, the amount of blood obtained for autologous blood reinfusion, and the gauze pad weight.

### Statistical methods

Statistical analysis was performed using SPSS 24.0 software. Normally distributed data were expressed as the mean ± standard deviation, x̅±s. Non-normally distributed data were expressed as the median (quartile), M (P25–P75). Count data was expressed by the frequency and rate.

## Results

### The clinical characteristics of the patients

The 48 women were 33 ± 5 years old, had 3.4 ± 1.4 pregnancies, had delivered 1.1 ± 0.6 times, and 34 cases (70.8%, 34/48) had a previous cesarean section. The number of previous cesarean sections was 0.9 ± 0.7, the preoperative hemoglobin level was 115 ± 12 g/L, the birth order was 0 to 2, and the gestational age at surgery was 36.5 ± 2.8 weeks. The patient’s age, gravidity, parity, previous cesarean section history, gestational age, current diagnosis, blood loss, and transfusion requirement are shown in Table S1[See Additional file 1].

### Intraoperative cases

#### Hemostasis during operation

The 48 cases of maternal hemostasis without hysterectomy were successful. Among these cases, there were 16 cases of pernicious placenta previa (33.3%, including one twin case), 11 cases of purely central placenta previa (22.9%, including one twin case), 18 cases of uterine scarring (37.5%, including two twin cases), one case of twin pregnancy (2.1%), one case of breech presentation (2.1%), and one case of pulmonary hypertension (2.1%).

#### Supplementary hemostasis scheme

An “8” suture combined with bilateral uterine and ovarian artery ligation was performed in one patient that had pernicious placenta previa with implantation. Five patients were treated with a Hayman suture, of which three cases had pernicious placenta previa, one case had central placenta previa, and one case had uterine scarring. Two patients were treated with an “8” suture for hemostasis, including one case of pernicious placenta previa and one case of central placenta previa.

#### Intraoperative blood loss

The average intraoperative blood loss in the 48 cases of maternal hemostasis was 865 ± 1176 ml. The maximum blood loss was 8000 ml and the minimum blood loss was 300 ml. Among the 48 cases, nine cases (18.8%, 8/48) had blood loss greater than 1000 ml, one of which was a case of twins with central placenta previa, and the remaining eight cases had pernicious placenta previa. Eleven cases were treated with blood transfusion products (22.9%, 11/48).

#### Surgery duration

Among the 48 operations, the shortest operation lasted 43 min and the longest operation lasted 162 min. The mean operation time was 77 ± 28 min. The King’s combined uterine suture operation time was 4.3 ± 0.8 min, accounting for 6% (4.3/77 min) of the total operation time.

### Postoperative situation

#### Recent recovery

Thirty patients (62.5%, 30/48) were observed in the OICU after surgery. There were no postpartum hemorrhages, or delayed postpartum hemorrhages, and no puerperal infections among the 48 cases. The postoperative hemoglobin level was 108 ± 12 g/L, and the length of postoperative hospital stay was 3.8 ± 1.1 days. No delayed postpartum hemorrhage occurred at discharge.

#### Ultrasound follow-up results

All 48 patients were followed up 42 days after delivery. Examination showed that the size of the uterus was normal and no other abnormalities were found.

#### Long-term prognosis

The longest follow-up period was 31 months and the shortest follow-up period was 5 months. Fourteen patients (29.2%, 14/48) underwent bilateral fallopian tube ligation at the same time, one of which was pregnant again 2 years after surgery and a healthy baby was delivered by caesarean section at 36 + 1 weeks of pregnancy. The return of menstruation time was 4.8 ± 2.3 months after delivery. Three patients did not have menstruation by the last visit due to lactation. No metrosynizesis and menstrual blood retention were found in any of the patients, and no complications were found, such as delayed postpartum hemorrhage and puerperal infection.

### Neonate conditions

In one of the 48 puerpera cases, the family members abandoned the rescue of the baby, because of the absence of the middle and far left part of the left forearm and the palm. The Apgar score was 9.5 ± 1.2 at 1 min and 9.9 ± 0.6 at 5 min. There were 15 cases (31.9%, 15/47) in which the neonate was transferred to the neonatal intensive care unit (NICU), of which nine cases (60%, 9/15) were referred for premature delivery with or without dyspnea before 34 + 5 weeks of pregnancy. Three of these cases were newborn twins and six cases (40%, 6/15) were born at 36 to 37 + 3 weeks of gestational age. The reason for NICU transfer was neonatal dyspnea and, after observation and improvement in the NICU, they were discharged from the hospital. No neonatal deaths occurred and all the neonates were well-developed at the follow-up that occurred up to half a year after birth.

## Discussion

The ACOG defines postpartum hemorrhage as blood loss of equal to or more than 1000 ml within 24 h postpartum, or with hypovolemic symptoms or signs of blood loss, including intrapartum bleeding [8]. Due to different etiologies and available treatment options, large differences in treatment methods exist between different patients. In general, a multidisciplinary and multifaceted treatment approach for postpartum hemorrhage should be adopted, including the identification and treatment of bleeding causes while maintaining hemodynamic stability [7, 9]. Uterine atony is still the most common cause of postpartum hemorrhage, accounting for about 70–80% of postpartum hemorrhages, and with increasing incidence [9–11]. Risk factors for postpartum hemorrhage during delivery include prolonged labor, labor induction, long-term use of oxytocin, chorioamnionitis, multiple pregnancies, polyhydramnios, and hysteromyoma. Therefore, early identification and management of high risk factors for postpartum hemorrhage can significantly reduce the incidence of postpartum hemorrhage. Despite this, about 1% of low-risk groups still have severe postpartum hemorrhage [7].

There are a variety of medications and surgical methods for the treatment of postpartum hemorrhage, but none have achieved consistent success. If first-line treatment (massage of the uterus, application of medicine for uterine contraction, etc.) fails to treat non-traumatic postpartum hemorrhage caused by uterine atony, surgical intervention must be considered to reduce maternal morbidity and mortality. Surgical treatments include uterine packing, uterine compression sutures, arterial ligation, and hysterectomy. Since it was first reported in 1997, the B-Lynch uterine compression suture has been the most popular and valuable suture technique and is suitable for postpartum hemorrhage caused by uterine atony during cesarean section [12]. Other uterine compression and suture techniques, such as the Cho and Hayman techniques, have also achieved good results that can reduce the incidence of emergency hysterectomy and can preserve patient fertility [13–22].

Since 2007, we have extensively applied the parauterine vascular ligation procedure for hemostasis during cesarean section and achieved good clinical outcomes. In 2009, Lin described and reported in detail that, after parauterine vascular ligation, some cases still exhibited diffuse errhysis in the lower part of the uterus in the long-term [23]. The reason for this may be that the blood supply from the ovarian communication branch was still present. In addition, the muscle tissue in the lower part of the uterus was thin and the contractility was poor. The cervix is mainly composed of connective tissue and there are fewer muscle fibers, providing a histological mechanism for the difficulty in controlling bleeding in the lower uterine segment and the internal cervix. When the placenta is attached to this part of the uterus, the placenta cannot peel off easily and the sinusoid cannot close easily after peeling of the placenta. This is the main cause of postpartum hemorrhage [2]. This commonly occurs in cases with uterine scarring, central placenta previa, pernicious placenta previa, etc.

According to Hayman et al., limitations of the B-Lynch uterine compression suture include the complexity of the surgical steps that are sometimes difficult for the surgeon to recall in emergency situations, as well as the inability of the sutures to completely cover the lower uterus [24]. In cases involving hemorrhaging of the lower uterine segment and the cervical internal opening, multi-point 8 stitches can be used for hemostasis. However, there is a risk that hemostasis cannot be achieved fast enough and the depth of the suture is not easy to grasp, which may penetrate the uterine muscle layer and adjacent organs, in which case gauze or balloon tamponade methods are often used, but are more tedious techniques.

Our King’s combined uterine suture is a conservative method for treating postpartum hemorrhage and has many advantages. First, the operation steps are simple and easy to grasp, shortening the operation time for quick and effective hemostasis and reducing blood loss of the patient. Second, the operation is performed before suture of the uterine incision, the hemostatic effect of the operation can be visually observed, and the hemostasis success rate is high. Our King’s combined uterine suture is especially suitable for the treatment of lower uterine segment hemorrhage during cesarean section, especially to treat and prevent pernicious placenta previa, uterine scarring, and lower uterine segment hemorrhage. All 48 patients were treated using the King’s combined uterine suture method to avoid postpartum hemorrhage or emergency hysterectomy, and to avoid any other surgical complications of hysterectomy in emergency situations. All cases recovered well and there were no complications associated with this method upon follow-up, such as intrauterine adhesions, irregular menstruation, or menstrual cramp-related pain. Postoperative follow-up with gynecological examination and B-ultrasound showed no uterine and pelvic abnormalities. However, when the posterior pelvic adhesion is closed, the lower segment of the posterior wall of the uterus can not be exposed, and the parauterine blood vessels cannot be ligated, King’s combined uterine suture can not be used to stop the bleeding quickly. It is common in patients with endometriosis and multiple abdominal and pelvic operations. At this time, the hemostatic method should be individualized, and generally can be used: ligation of the communicating branch of uterine and ovarian artery, hemostasis of the anterior and posterior wall of the lower segment of the uterus respectively, 8-shaped suture of the bleeding point, and so on.

The possible hemostatic mechanism of the King’s combined uterine suture is as follows: parauterine vessel ligation blocks the main blood supply to the uterus and slows down uterine blood flow. Longitudinal suture of the lower uterine segment blocks the arcuate artery regional blood supply, further reducing blood flow in the lower uterus and the cervical internal opening and promoting thrombus formation in the wound to achieve hemostasis. For successful surgery, it is key to adequately separate and expose the surgical approach, including adhesions between the uterus, abdominal wall, intestine, omentum, and adhesions between the bladder and lower uterus.

In summary, King’s combined uterine suture is a safe, rapid, and effective method for uterine hemostasis during cesarean section, especially in cases with pernicious placenta previa, central placenta previa, and uterine scarring, as well as twin pregnancies, lower uterine trauma caused by difficulty in taking the fetal head, and other causes of extensive bleeding in the lower uterus. The King’s combined uterine suture can achieve rapid hemostasis, reduce blood loss, and retain the fertility of young women. This procedure is a valuable operation method; however, further evaluation is required for subsequent pregnancies and other situations.

## Supplementary Information

**Additional file 1: Table S1.** Demography and the clinical outcomes for these 48 patients of this series.

**Additional file 2.**

## Data Availability

All data generated or analysed during this study are included in this published article [and its supplementary information files].

## References

[1] Dahlke JD, Mendez-Figueroa H, Maggio L, Hauspurg AK, Sperling JD, Chauhan SP, et al. Prevention and management of postpartum hemorrhage: a comparison of 4 national guidelines. Am J Obstet Gynecol. 2015. 10.1016/j.ajog.2015.02.023. 10.1016/j.ajog.2015.02.023 25731692

[2] Xing Xie TD. Beihua Kong, obstetrics and gynecology 9th. Chaoyang: People’s Medical Publishing House; 2018.

[3] Say L, Chou D, Gemmill A, Tunçalp Ö, Moller AB, Daniels J, et al. Global causes of maternal death: a WHO systematic analysis. Lancet Glob Health. 2014. 10.1016/S2214-109X(14)70227-X. 10.1016/S2214-109X(14)70227-X 25103301

[4] Weeks AD. Secondary prevention: a new era for postpartum haemorrhage? BJOG An Int J Obstet Gynaecol. 2016. 10.1111/1471-0528.13606. 10.1111/1471-0528.13606 26412462

[5] Petersen EE, Davis NL, Goodman D, Cox S, Mayes N, Johnston E, et al. Vital signs: pregnancy-related deaths, United States, 2011–2015, and strategies for prevention, 13 states, 2013–2017. MMWR Morb Mortal Wkly Rep. 2019. 10.15585/mmwr.mm6818e1. 10.15585/mmwr.mm6818e1 PMC6542194 31071074

[6] Mavrides E, Allard S, Chandraharan E, Collins P, Green L, Hunt BJ, Riris S, Thomson AJ on behalf of the Royal College of Physicians. Prevention and management of postpartum haemorrhage. BJOG. 2016. 10.1111/1471-0528.14178.

[7] Bulletins-Obstetrics C . Practice Bulletin No. 183: Postpartum Hemorrhage[J]. Obstet Gynecol. 2017;130. 10.1097/AOG.0000000000002351. 10.1097/AOG.0000000000002351 28937571

[8] Menard MK, Main EK, Currigan SM. Executive summary of the reVITALize initiative: standardizing obstetric data definitions. Obstet Gynecol. 2014. 10.1097/AOG.0000000000000322. 10.1097/AOG.0000000000000322 24901267

[9] Joseph KS, Rouleau J, Kramer MS, Young DC, Liston RM, Baskett TF. Investigation of an increase in postpartum haemorrhage in Canada. BJOG An Int J Obstet Gynaecol. 2007. 10.1111/j.1471-0528.2007.01316.x. 10.1111/j.1471-0528.2007.01316.x 17516968

[10] Bateman BT, Berman MF, Riley LE, Leffert LR. The epidemiology of postpartum hemorrhage in a large, nationwide sample of deliveries. Anesth Analg. 2010. 10.1213/ANE.0b013e3181d74898. 10.1213/ANE.0b013e3181d74898 20237047

[11] Kramer MS, Berg C, Abenhaim H, Dahhou M, Rouleau J, Mehrabadi A, et al. Incidence, risk factors, and temporal trends in severe postpartum hemorrhage. Am J Obstet Gynecol. 2013. 10.1016/j.ajog.2013.07.007. 10.1016/j.ajog.2013.07.007 23871950

[12] B-Lynch C, Coker A, Lawal AH, Abu J, Cowen MJ. The B-Lynch surgical technique for the control of massive postpartum haemorrhage: an alternative to hysterectomy? Five cases reported. BJOG An Int J Obstet Gynaecol. 1997. 10.1111/j.1471-0528.1997.tb11471.x. 10.1111/j.1471-0528.1997.tb11471.x 9091019

[13] Cho JH, Jun HS, Lee CN. Hemostatic suturing technique for uterine bleeding during cesarean delivery. Obstet Gynecol. 2000. 10.1016/S0029-7844(00)00852-8. 10.1016/s0029-7844(00)00852-8 10928901

[14] Ouahba J, Piketty M, Huel C, Azarian M, Feraud O, Luton D, et al. Uterine compression sutures for postpartum bleeding with uterine atony. BJOG An Int J Obstet Gynaecol. 2007. 10.1111/j.1471-0528.2007.01272.x. 10.1111/j.1471-0528.2007.01272.x 17355361

[15] Hwu YM, Chen CP, Chen HS, Su TH. Parallel vertical compression sutures: a technique to control bleeding from placenta praevia or accreta during caesarean section. BJOG An Int J Obstet Gynaecol. 2005. 10.1111/j.1471-0528.2005.00666.x. 10.1111/j.1471-0528.2005.00666.x 16167948

[16] Matsubara S, Yano H, Taneichi A, Suzuki M. Uterine compression suture against impending recurrence of uterine inversion immediately after laparotomy repositioning. J Obstet Gynaecol Res. 2009. 10.1111/j.1447-0756.2008.01011.x. 10.1111/j.1447-0756.2008.01011.x 19751352

[17] Matsubara S, Yano H, Ohkuchi A, Kuwata T, Usui R, Suzuki M. Uterine compression sutures for postpartum hemorrhage: an overview. Acta Obstet Gynecol Scand. 2013. 10.1111/aogs.12077. 10.1111/aogs.12077 23330882

[18] Matsubara S, Kuwata T, Baba Y, Usui R, Suzuki H, Takahashi H, et al. A novel “uterine sandwich” for haemorrhage at caesarean section for placenta praevia. Aust New Zeal J Obstet Gynaecol. 2014. 10.1111/ajo.12184. 10.1111/ajo.12184 24506478

[19] Zhang ZW, Liu CY, Yu N, Guo W. Removable uterine compression sutures for postpartum haemorrhage. BJOG An Int J Obstet Gynaecol. 2015. 10.1111/1471-0528.13025. 10.1111/1471-0528.13025 25175111

[20] Li GT, Li XF, Liu YJ, Li W, Xu HM. Symbol ‘“&”’ suture to control atonic postpartum hemorrhage with placenta previa accreta. Arch Gynecol Obstet. 2015. 10.1007/s00404-014-3502-3. 10.1007/s00404-014-3502-3 25288270

[21] Mohamed MA, Mohammed AH. Parallel vertical compression sutures to control bleeding in cases of placenta previa and accreta. J Matern Neonatal Med. 2019. 10.1080/14767058.2017.1387895. 10.1080/14767058.2017.1387895 29034750

[22] Shih JC, Liu KL, Kang J, Yang JH, Lin MW, Yu CU. ‘Nausicaa’ compression suture: a simple and effective alternative to hysterectomy in placenta accreta spectrum and other causes of severe postpartum haemorrhage. BJOG An Int J Obstet Gynaecol. 2019. 10.1111/1471-0528.15410. 10.1111/1471-0528.15410 PMC6585672 30009547

[23] Lin J. Application of parauterine vascular ligation in hemostasis of placenta previa during cesarean section. Strait J Prev Med. 2009;15(6):83–84.

[24] Hayman RG, Arulkumaran S, Steer PJ. Uterine compression sutures: surgical management of postpartum hemorrhage. Obstet Gynecol. 2002. 10.1016/S0029-7844(01)01643-X. 10.1016/s0029-7844(01)01643-x 11864681

