# Supplementary material for: Study on the application of King’s combined uterine suture for hemostasis during cesarean section
Source: BMC Pregnancy Childbirth. 2021 Nov 10;21:762. doi: 10.1186/s12884-021-04231-4 (PMC8579605; doi:10.1186/s12884-021-04231-4)
Supplement: Supplementary file 1 — Additional file 1: Table S1. Demography and the clinical outcomes for these 48 patients of this series. [file 12884_2021_4231_MOESM1_ESM.pdf]

Table S1. Demography and the clinical outcomes for these 48 patients of this series

Case No.	age	Gravidity parity	Previous CS	GA of birth (w)	Presenting diagnosis	Pre-op Hb (g/dL)	Estimated blood loss (mL)	Post-op Hb (g/dL)	Blood transfusion (u)	Operation time/King's combined uterine suture (min)	Adjunctive hemostatic procedures	Resumed menstruation/got gestation (months)	Fellow-up (months)
1	27	G_1P_0	0	39	Complete placenta previa	114	600	102	0	60/5	Hayman	1	19
2	30	G_3P_1	1	38^{+6}	Previous CS scar	106	500	99	0	74/4		7	18
3	32	G_3P_1	0	36^{+6}	Complete placenta previa	134	600	126	0	76/4		6	17
4	31	G_4P_1	0	36^{+4}	Complete placenta previa	122	400	126	0	43/3		6	15
5	34	G_3P_1	1	34^{+4}	Pernicious placenta previa; Placenta accreta	103	1600	123	338ml Autologous transfusion; 2U PRBC; 400ml FFP	112/6		6	19
6	35	G_2P_1	1	36	Pernicious placenta previa; Placenta accreta	105	8000	88	436ml Autologous transfusion; 8U PRBC; 800ml FFP	162/6	Hayman	12	18
7	41	G_3P_1	1	37^{+6}	Previous CS scar	114	600	102	0	83/4		6	16
8	28	G_2P_1	0	37^{+4}	Complete placenta previa	124	650	110	0	82/4		4	17
9	37	G_3P_1	1	36^{+4}	Monochorionic-	124	400	115	0	48/3		6	15

					dimaniotic; Previous CS scar								
10	35	G_4P_2	2	37^{+4}	Previous CS scar	132	400	127	0	63/4		2	13
11	27	G_5P_1	1	38^{+5}	Previous CS scar	102	400	97	0	43/4		6/24	29
12	28	G_2P_1	1	23^{+4}	Pernicious placenta previa; Placenta accreta	104	2300	89	4U PRBC	111/5		2	8
13	37	G_3P_1	1	34^{+5}	Dichorionic–diamniotic ;Previous CS scar	151	500	138	0	63/4		2	17
14	34	G_4P_2	2	31^{+3}	Previous CS scar	107	600	96	0	75/5		2	15
15	36	G_5P_1	0	38^{+2}	Breech presentation	123	400	93	0	43/4		1	7
16	36	G_5P_1	1	31^{+5}	Pernicious placenta previa; Placenta accreta	108	600	126	4U PRBC	65/5		3	17
17	33	G_2P_1	1	39^{+1}	Previous CS scar; Uterine myoma	135	600	103	0	77/5		6	15
18	24	G_3P_1	1	37^{+1}	Pernicious placenta previa	110	500	105	0	59/4		8	31
19	38	G_3P_1	1	37^{+6}	Pernicious placenta previa	111	600	100	0	64/4		10	29
20	36	G_4P_0	0	33^{+1}	Complete placenta previa; Placenta accreta	117	600	103	0	81/5		2	25
21	32	G_4P_1	1	34^{+3}	Pernicious	107	800	112	0	107/5		6	25

					placenta previa;Preeclampsia								
22	30	G_5P_2	2	34	Pernicious placenta previa; Placenta accreta; ;Monochorionic-dimaniotic;	100	2800	114	810ml Autologous transfusion; 6U PRBC; 800ml FFP	145/6	Hayman	4	15
23	37	G_3P_2	2	37^{+6}	Previous CS scar	112	400	99	0	60/4		6	15
24	33	G_3P_1	0	37	Complete placenta previa	106	500	107	0	57/4		6	12
25	28	G_5P_2	2	37^{+1}	Pernicious placenta previa	97	400	92	0	56/4		2	11
26	34	G_5P_1	1	39^{+3}	Pernicious placenta previa; Placenta accreta	126	1200	106	300ml Autologous transfusion	96/5		3	26
27	27	G_1P_0	0	37^{+3}	Complete placenta previa; Placenta accreta	113	500	108	0	75/5		5	25
28	34	G_6P_1	2	38^{+3}	Previous CS scar	106	400	96	0	72/4		4	22
29	31	G_2P_1	0	36^{+5}	Complete placenta previa	106	600	100	0	53/4	Hayman	6	13
30	37	G_3P_2	2	39	Previous CS scar	120	400	111	0	70/4		4	13
31	26	G_4P_1	1	37	Pernicious placenta previa;	116	800	117	2U PRBC	78/5		5	12

					Placenta accreta								
32	34	G_2P_1	0	37^{+2}	Complete placenta previa	127	400	124	0	73/4		10	7
33	30	G_2P_0	0	37^{+3}	Dichorionic – diamniotic	129	500	104	0	60/4		6	23
34	24	G_4P_1	1	37	Pernicious placenta previa	108	600	102	0	85/5	Hyman	2	17
35	32	G_3P_1	1	37	Pernicious placenta previa; Placenta accreta	113	400	111	0	76/4		1	10
36	38	G_3P_1	1	38^{+6}	Previous CS scar	100	350	91	0	65/4		6	10
37	29	G_3P_1	1	36	Pernicious placenta previa; Placenta accreta	106	2100	110	240ml Autologous transfusion; 8U PRBC; 400ml FFP	130/5	Ligation of communicating branches of bilateral uterine and ovarian arteries; Figure eight suture	4	8
38	36	G_2P_0	0	37^{+5}	Complete placenta previa;Uterine myoma	125	600	112	0	63/4		4	7

39	29	G_3P_1	1	39^{+4}	Previous CS scar	124	500	119	0	47/3	Hayman	7	8
40	30	G_2P_1	1	34^{+2}	Pernicious placenta previa; Placenta accreta	108	1100	120	4U PRBC;400ml FFP	110/5		10	26
41	31	G_2P_1	1	38^{+4}	Previous CS scar	96	500	94	0	51/3		6	18
42	30	G_6P_2	2	36^{+1}	Previous CS scar	95	600	89	0	90/5		4	9
43	31	G1P0	0	32+5	Complete placenta previa; Dichorionic – diamniotic	106	1200	101	4U PRBC; 400ml FFP	97/5	Figure eight suture	6	7
44	34	G5P1	0	38+2	Breech presentation	139	400	123	0	49/3		4	7
45	25	G2P1	0	37+5	Pulmonary hypertension	115	400	112	0	56/3		Lactation without menstruation	6
46	45	G7P2	1	36+4	Pernicious placenta previa	125	1500	98	4U PRBC; 400ml FFP	155/5	Figure eight suture	3	6
47	41	G6P2	1	39+1	Previous CS scar	124	300	120	0	61/4		6	6
48	37	G3P1	1	39+2	Previous CS scar	128	400	109	0	66/4		Lactation without menstruation	5

Case No.	Admission to OICU	How many days after discharge	Neonatal weight (g)	Apgar scores (1min)	Apgar scores (5min)	Admission to NICU	Reasons for admission to NICU
1	Yes	3	3045	10	10	No	
2	No	4	3215	10	10	No	
3	Yes	4	3180	10	10	No	
4	Yes	3	2820	10	10	No	
5	Yes	6	2240	7	10	Yes	Premature; Neonatal dyspnea
6	Yes	8	2900	7	10	Yes	Neonatal dyspnea
7	Yes	3	3545	10	10	No	
8	Yes	3	3120	10	10	No	
9	No	4	2865/2690	10	10/10	No/No	
10	No	4	3015	10	10	No	
11	No	4	3650	10	10	No	
12	Yes	6	650				The fetal left forearm and palm were missing, and the family gave up the rescue.
13	Yes	3	1965/2350	10/10	10/10	Yes/Yes	Premature; Neonatal dyspnea
14	No	4	1655	10	10	Yes	Premature; Neonatal dyspnea
15	Yes	3	3520	10	10	No	
16	Yes	5	1690	8	10	Yes	Premature; Neonatal dyspnea
17	No	3	4130	10	10	No	
18	Yes	4	3080	9	10	Yes	Neonatal dyspnea
19	Yes	6	3175	10	10	No	
20	Yes	4	2220	10	10	Yes	Premature

21	Yes	4	2340	10	10	Yes	Premature; Neonatal dyspnea
22	Yes	7	1940/1600	4/3	6/6	Yes/Yes	Premature; Neonatal dyspnea
23	No	4	3380	10	10	No	
24	Yes	3	2550	10	10	No	
25	Yes	4	2850	10	10	No	
26	Yes	4	3100	10	10	No	
27	Yes	4	2640	7	10	Yes	Neonatal dyspnea
28	No	3	3700	10	10	No	
29	No	3	3260	10	10	Yes	Neonatal dyspnea
30	No	3	3625	10	10	No	
31	Yes	3	2870	10	10	Yes	Neonatal dyspnea
32	Yes	3	2990	10	10	No	
33	Yes	4	2230/2720	8/8	10/10	No/No	
34	Yes	3	3260	8	10	No	
35	Yes	3	2620	10	10	No	
36	No	3	3160	10	10	No	
37	Yes	5	3160	10	10	No	
38	Yes	4	2850	10	10	No	
39	No	3	3010	10	10	No	
40	Yes	3	2230	8	10	Yes	Premature; Neonatal dyspnea
41	No	4	2865	10	10	No	
42	No	4	3050	10	10	Yes	Neonatal dyspnea
43	Yes	4	1820/1505	10/10	10/10	Yes/Yes	Premature; Neonatal dyspnea
44	No	3	3160	10	10	No	
45	No	3	3100	10	10	No	

46	Yes	3	2260	10	10	No	
47	No	3	3335	10	10	No	
48	No	3	2950	10	10	No	

No: number; CS: cesarean section, GA: gestational age(week); Pre-op, Post-op: pre-operational, post-operational; Hb: hemoglobin; PRBC: packed red blood cells; FFP: fresh-frozen plasma; U: unit; OICU: Obstetric intensive care unit; NICU:Neonatal Intensive Care Unit.
