# Supplementary material for: Study on the application of King’s combined uterine suture for hemostasis during cesarean section
Source: BMC Pregnancy Childbirth. 2021 Nov 10;21:762. doi: 10.1186/s12884-021-04231-4 (PMC8579605; doi:10.1186/s12884-021-04231-4)
Supplement: Supplementary file 2 — Additional file 2. [file 12884_2021_4231_MOESM2_ESM.zip › Demonstration of the operation process of King's combined uterine suture.docx]

Demonstration of the operation process of King's combined uterine suture(Please watch the video for specific steps):

1. After placental expulsion, the uterus was lifted out of the abdominal incision, followed by the ligation of left parauterine vessels.

2.The right parauterine vessel was ligated in the same way.

3.Delivering the placenta.

4.Based on the parauterine vascular ligation,longitudinal suture of the left lower uterine segment was performed.

5.Longitudinal suture of the right lower uterine segment was performed
